# Exploring the association between ultra-processed foods and COPD: a case-control study

**DOI:** 10.1186/s12890-024-02903-3

**Published:** 2024-03-08

**Authors:** Zahra Salehi, Hanieh Malmir, Batoul Ghosn, Shokouh Onvani, Mohammad Emami Ardestani, Awat Feizi, Ahmad Esmaillzadeh, Leila Azadbakht

**Affiliations:** 1 Department of Community Nutrition, School of Nutritional Sciences and Dietetics, Tehran University of Medical Sciences, Tehran, P.O. Box 14155-6117, Iran; 2 Obesity and Eating Habits Research Center, Endocrinology and Metabolism Molecular Cellular Sciences Institute, Tehran University of Medical Sciences, Tehran, Iran; 3 Food Security Research Center, Isfahan University of Medical Sciences, Isfahan, Iran; 4 Department of Community Nutrition, School of Nutrition and Food Science, Isfahan University of Medical Sciences, Isfahan, Iran; 5 Division of Pulmonology, Department of Internal Medicine, School of Medicine, Isfahan University of Medical Sciences, Isfahan, Iran; 6 Department of Epidemiology and Biostatistics, Endocrinology and Metabolism Research Center, Isfahan University of Medical Sciences, Isfahan, Iran; 7 Department of Community Nutrition, School of Nutritional Science and Dietetics, Tehran University of Medical Sciences, Tehran, Iran; 8 Diabetes Research Center, Endocrinology and Metabolism Clinical Sciences Institute, Tehran University of Medical Sciences, Tehran, Iran

**Keywords:** Ultra-processed foods, Chronic obstructive pulmonary disease, Diet, Case-control

## Abstract

**Background:**

While it is known that the overconsumption of ultra-processed foods (UPFs) is associated with a heightened risk of respiratory ailments, the specific effects of UPF intake on COPD remain unclear. This study was designed to explore the potential link between COPD and the consumption of UPFs among adult individuals in Iran.

**Methods:**

In this hospital-based case-control study conducted at Alzahra University Hospital in Isfahan, Iran, we enrolled 84 patients newly diagnosed with COPD, along with 252 healthy controls matched for age and sex. COPD was defined based on the results of spirometry tests, specifically when the forced expiratory volume per second (FEV1) was less than 80% or the ratio of FEV1 to forced vital capacity (FVC) was less than 70%. To evaluate the dietary intake of the participants, we utilized a validated food frequency questionnaire (FFQ) consisting of 168 items. Additionally, we gathered data on potential confounding factors using a pre-tested questionnaire.

**Results:**

The mean ages for the case and control groups were 57.07 and 55.05 years, respectively. Our study found no significant association between the intake of ultra-processed foods (UPFs) and the likelihood of COPD, with an odds ratio (OR: 0.78, 95% CI: 0.34–1.77). This lack of association persisted even after adjusting for factors such as energy intake, sex, and age (OR: 0.48; 95% CI: 0.19–1.21). Further controlling for potential confounders like body mass index (BMI), physical activity, and smoking status did not alter this finding (OR: 0.367; 95% CI: 0.123–1.1008, *P* = 0.074).

**Conclusions:**

In our study, we observed no significant association between the intake of Ultra-Processed Foods (UPFs) and the odds of Chronic Obstructive Pulmonary Disease (COPD). This finding remained consistent even after adjusting for factors such as energy intake, sex, age, Body Mass Index (BMI), physical activity, and smoking status. Therefore, within the scope of our study, it appears that the consumption of UPFs does not significantly impact the likelihood of developing COPD. However, we recommend further research to deepen our understanding of the intricate relationship between dietary habits and respiratory health.

## Introduction

Chronic Obstructive Pulmonary Disease (COPD) is a significant global health concern. According to the Global Burden of Disease Study 2019, COPD accounted for a substantial number of deaths and disability-adjusted life years (DALYs) worldwide [1]. The study reported that in 2019, there were 212.3 million prevalent cases of COPD globally, with COPD accounting for 3.3 million deaths and 74.4 million DALYs [1]. Another study found that the total number of COPD cases increased by 39.5% from 1990 to 2017 [2].

The importance of diet in managing and preventing COPD has been increasingly recognized. A balanced diet rich in antioxidants and anti-inflammatory foods may help prevent and manage COPD [3, 4]. In particular, the consumption of ultra-processed foods has been linked to a higher risk of COPD [5]. These foods often contain high levels of added sugar, fat, and/or salt, but lack vitamins and fiber [6]. A study found that elevated consumption of ultra-processed food was associated with a higher risk of COPD, and this association was primarily mediated by glucose, inflammation, and lipids [5]. Conversely, substituting unprocessed or minimally processed food for ultra-processed food was associated with a decreased risk of COPD [5]. Therefore, maintaining a healthy diet and avoiding ultra-processed foods could play a crucial role in managing and preventing COPD. However, more research is needed to further understand these relationships and to develop dietary guidelines for individuals with COPD.

COPD is a leading cause of death, illness, and healthcare load worldwide [7], and is characterized by chronic bronchitis, small airway blockage, and emphysema, leading to chronic inflammation of the airways and pulmonary parenchyma with irreversible and progressive airflow restriction [7]. With 210 million COPD patients worldwide and projections to become the third leading cause of mortality by 2030 [8], understanding the factors contributing to COPD is of paramount importance.

While smoking is the most significant cause of COPD [9], but factors such as workplace pollution, environmental pollution and genetics are also important contributors in the pathogenesis of COPD [10]. Recent studies have underscored the significance of dietary habits in both the onset and progression of chronic diseases [11]. Recent studies have underscored the significance of dietary habits in both the onset and progression of chronic diseases, including COPD [12–14].

Over the past decades, diets in many countries have shifted towards a significant increase in the consumption of ultra-processed foods (UPFs) [15–18],which are high in additives, salt, free sugars, fats, preservatives, synthetic antioxidants [19]. but often lack important micronutrients, fiber, protein, and phytochemicals [20]. The potential negative effects of these ingredients on our health warrant further research. Existing research has explored the relationship between processed meat consumption and the incidence of Chronic Obstructive Pulmonary Disease (COPD) where a systematic review and meta-analysis of prospective cohort studies and found a positive association between processed red meat intake and the risk of COPD [21]. A study found that regular meat consumption, including processed meat, is associated with a range of diseases, including heart disease, pneumonia, and diabetes but did not find a significant association between processed meat consumption and the risk of COPD [22]. Also a systematic review and meta-analysis of prospective cohort studies found that higher consumption of red meat and processed meat was associated with an increased risk of colorectal cancer [23]. While this study did not specifically investigate the relationship between processed meat consumption and COPD, it contributes to the broader understanding of the potential health impacts of processed meat consumption. This represents a significant gap in the current body of research and underscores the importance of our study in contributing to this area of knowledge.

While the effects of UPFs on several chronic diseases such as obesity, metabolic syndrome, diabetes, hypertension, dyslipidemia, heart disease, and cancer have been previously studied [24–31], their impact on COPD remains under-investigated. UPFs typically have poor nutritional profiles, are hyper-palatable, and contain biologically harmful compounds, all of which can negatively impact health [32]. In the context of Chronic Obstructive Pulmonary Disease (COPD), it is plausible that the consumption of UPFs could contribute to the development and progression of the disease. For instance, the high levels of free sugars, saturated fats, and sodium found in UPFs could lead to systemic inflammation, a key factor in the pathogenesis of COPD [32, 33]. Furthermore, the additives and preservatives commonly used in UPFs could potentially have a direct detrimental effect on lung function [33]. However, the exact mechanisms linking UPF consumption and COPD are not yet fully understood, and more research is needed in this area. It’s also important to note that while UPF consumption could potentially contribute to COPD, it is likely just one of many factors involved, alongside others such as smoking, air pollution, and genetic predisposition [32].

Therefore, this study aims to investigate the relationship between UPF consumption and the incidence of COPD, contributing to the limited body of research in this area. Our findings provide valuable insights into the potential role of diet, specifically UPF consumption, in the development and progression of COPD.

## Materials and methods

### Participants

The current case-control study was conducted on 84 COPD patients who were recently diagnosed, and 252 controls in the period between 2015 and 2016 at Al-Zahra Hospital, University of Isfahan, Iran. Cases were patients with forced expiratory volume (FEV1) < 80 or FEV1/ forced vital capacity (FVC) < 70%. The participants were all above the age of 30 and had received their COPD diagnosis from a pulmonologist. The diagnostic criteria were based on spirometry test results, a common method for assessing lung function [34]. A patient was identified as having COPD if their ratio of forced expiratory volume in 1 s (FEV1) to forced vital capacity (FVC) was below 70%, or if their FEV1 was less than 80% of the predicted value. The control group was made up of 252 healthy individuals from the same hospital, none of whom had a history of COPD. Both cases and controls were paired based on age (within a 5-year range) and gender. The control subjects were those who visited the same hospital’s outpatient clinics and were enlisted concurrently with the cases. The criteria for including a subject in the case group was a COPD diagnosis confirmed by a physician and a spirometry test. We excluded participants from the study if they had a dementia, stroke, or any condition that would prevent them from participating in an interview. In addition, other chronic diseases such as severe heart failure, chronic liver cirrhosis, inflammatory bowel disease, renal failure, uncontrolled thyroid disease, rheumatoid arthritis, cancer in the last 3 years, a systematic course of long-term treatment with steroid drugs, chronic infections (HIV, tuberculosis, etc.), cachexia, and other lung problems like fibrosis were considered as non-inclusion criteria.

### Dietary intakes assessment

A validated 168-item FFQ was used to assess participants’ usual dietary intake during the last year. The reliability and validity of the FFQ have been confirmed in previous studies [35]. This FFQ included of 168 food items which each food item has a standard serving size [36]. A trained interviewer conducted face-to-face interviews to complete the FFQ. Household measures were used to convert reported consumptions to grams per day [37]. Daily energy and nutrient intakes for each person were then calculated using the food composition database of the US Department of Agriculture, which was modified for Iranian foods [38].

According to NOVA classification system which was suggested by Monteiro et al. [39], ultra-processed foods were selected. The NOVA classification system classifies foods into 4 different categories: the first category includes unprocessed and slightly processed foods, including foods that are fresh or processed without any additives such as salt, sugar, fats or oils. The second category includes processed cooking ingredients such as fats, sugar, or salt, consumed in mixture with unprocessed and minimally processed foods in meal preparation. The third group includes processed foods such as canned fruits or vegetables, salted nuts, cured, or smoked meats, and cheese which are produced by adding processed culinary ingredients to unprocessed or least processed foods. The fourth category includes ultra-processed foods, such as ice cream, biscuits, cakes, chocolates, chips, pastries, jams, confectionery, sugar added breakfast cereals, crisps, high caloric snack products, sauces, fruit and milk beverages with added sugar, cereal bars, sweetened and non-caloric soft drinks, and pasta, poultry, fish meat and vegetable dishes, hot dogs, burgers, chicken nuggets fish sticks, sausages pre-prepared pizza, processed soups and noodles, infant formulas and baby food products. These products have undergone the highest level of processing. In the current study, only foods in the fourth group (ultra-processed foods) were analyzed.

In our methodology, we utilized the NOVA classification system to categorize food items, focusing specifically on ultra-processed foods. Each food item was classified as either an ultra-processed food or not, providing us with nominal data. To further refine our analysis, we divided the consumption of ultra-processed foods into quintiles, thereby presenting our data on an ordinal scale. This approach allowed us to examine the relationship between varying levels of ultra-processed food consumption and the risk of COPD in a more nuanced manner.

### Ultra processed food score construction

In our study, we evaluated the overall dietary quality by examining both food groups and nutrients across quintiles of the ultra-processed food score. This involved dividing the participants into five distinct groups based on their ultra-processed food consumption. Each group represented a fifth, or 20%, of the data. To create the ultra-processed food score, we used food frequency questionnaires to collect data on the participants’ dietary habits. Each food item was then classified according to the NOVA classification, and the proportion of ultra-processed foods in the diet was calculated. This gave us the ultra-processed food score for each participant. Next, we divided these scores into quintiles. This was done by sorting the scores from lowest to highest and then dividing them into five equal groups. Each quintile thus represents a range of ultra-processed food scores, allowing us to compare the dietary quality across different levels of ultra-processed food consumption.

### Pulmonary function assessment

A trained technician used spirometry test to evaluate pulmonary function and computed FVC, FEV1, and FEV1 / FVC. The reliability and validity of the spirometry have been confirmed in previous studies [34]. Evaluation of other respiratory signs, such as persistent cough, sputum production and pain intensity were also assessed. In the current study, persistent cough was defined as cough for more than three weeks [40], while sputum production was defined as the production of sputum for more than three months in two successive years [41, 42]. The visual analog scale was used to assess pain intensity. The Visual Analog Scale is a 100 mm horizontal line used by patients to quantify their pain intensity. One end represents the absence of pain, while the opposite end signifies the most severe pain experienced [43].

### Other variables assessment

Through a comprehensive questionnaire, we gathered data on participants’ age, gender, education level, marital status, smoking habits (categorized as never smoker, current smoker, or former smoker), medication and supplement usage, and family history of lung disorders. Participants’ height was measured in a standard upright position without shoes, while body weight was recorded to the nearest 100 g using a digital scale, with participants wearing minimal clothing. These measurements were then used to calculate the Body Mass Index (BMI) by dividing weight (in kilograms) by the square of height (in meters). To assess participants’ daily physical activity, we utilized the long form of the International Physical Activity Questionnaire (IPAQ) [43]. This assessment considered the number of days per week and the duration (in minutes) of physical activity per day. MET minutes of these activities was quantified using the Metabolic Equivalent for Task (MET) and expressed as MET-minutes per week.

### Statistical methods

In the current study, classification of participants across quintiles of ultra-processed food intakes was done. The study participants’ general characteristics and dietary intakes were analyzed and compared across different levels of UPF score using t-test and ANOVA test for continuous variables and chi-square tests for categorical variables. Logistic regression was used to investigate the association between UPF and COPD in several models. Model 1 was adjusted for energy intake (kcal/d), gender, and age. Model II was further adjusted for BMI, physical activity, and smoking status. All confounders were selected based on previous publications [44, 45]. To evaluate the normality of our data, we employed the Shapiro-Wilk test and conducted a visual examination of histograms and Q-Q plots. This comprehensive approach allowed us to accurately assess the distribution of our data. The statistical analyses were carried out using SPSS version 21. P-values were considered significant at *P* < 0.05.

## Results

General characteristics of study participants were reported in Table 1. Cases were more likely to be smokers, married, employed, and having a low level of physical activity, lung disease family history, and air pollution exposure compared to controls. As expected, cases were more likely to have lower FVC, FEV1, and FEV1 / FVC ratios compared to controls.

**Table 1:** Comparative Characteristics of study participants of COPD patients versus control subjects

	Cases (*n* = 84)		Control (*n* = 252)		
	%	Mean + SD	%	Mean + SD	P value
Age (Year)		57.07 ± 12.47		55.05 ± 12.34	0.197
Gender (Male)	89.3		89.3		0.999
Marital Status	90.5		87.6		0.030
University Education	6		9.1		0.362
Employment status	21.4		12.9		< 0.0001
BMI		25.6 ± 4.8		25.9 ± 3.8	0.550
Physical activity (MET/week)		5285 ± 8097		11,759 ± 6307	< 0.0001
History of pulmonary disease	39.3		19.8		0.006
Habitat Air Pollution					< 0.0001
Urban	41.7		78		
Rural	41.7		13.9		
Industrial	16.7		8.1		
Hookah use	100		39.5		< 0.0001
Cigarette smoking					< 0.0001
Current smoker	57.1		44.8		
Former smoker	0.0		22.0		
Never smoker	31.0		7.8		
Smoke exposure	16.7		12.3		0.431
FEV1		55.2 ± 18.6		95.0 ± 12.5	< 0.0001
FVC		71.2 ± 17.9		92.6 ± 13.3	< 0.0001
FEV1/FVC		62.7 ± 9.3		82.5 ± 6.1	< 0.0001

COPD = chronic obstructive pulmonary disease, BMI = body mass index, FEV1 = forced expiratory volume in 1 s, FVC = forced volume capacity

The P-values were determined using t-test for continuous variables and the Chi-square test for categorical variables

Dietary intakes of study participants were reported in Table 2. Cases consumed less red and processed meats, whole grains, and sugar-sweetened beverages compared to controls. Furthermore, compared to controls, cases had higher energy, carbohydrates, magnesium, sodium, vitamin K, cholesterol, and dietary fiber.

**Table 2:** Comparative analysis of mean dietary intakes among COPD patients and control subjects

	Cases (*n* = 84)	Control (*n* = 252)	
	Mean + SD	Mean + SD	P value
Energy (kcal/d)	2882 ± 781	2624 ± 682	0.004
**Food groups (g/d)**			
Whole grains	49.3 ± 91.9	125.8 ± 115.9	< 0.0001
Fruit	362.8 ± 214.1	368.7 ± 180.4	0.811
Vegetables	352.9 ± 151.0	340.1 ± 173.6	0.554
Low-fat dairy products	397.6 ± 237.6	352.1 ± 170.6	0.065
Legume and nuts	43.9 ± 38.2	46.9 ± 28.6	0.458
Red meat/processed meat	69.8 ± 37.9	53.6 ± 41.3	0.002
Sugar-sweetened beverages	45.9 ± 76.7	63.5 ± 71.3	0.061
Ultra-processed foods	132.03 ± 128.2	132.6 ± 87.6	0.96
**Macronutrients (g/d)**			
Carbohydrate	462 ± 137	415 ± 128	0.005
Protein	95 ± 25	110 ± 159	0.390
Fat	78 ± 31	70 ± 35	0.087
Dietary fiber	19 ± 7	22 ± 10	0.021
Sodium (mg/d)	3565 ± 1108	4308 ± 1570	< 0.0001
Magnesium (mg/d)	309 ± 106	429 ± 154	< 0.0001
Potassium (mg/d)	3901 ± 1324	4299 ± 2709	0.197
Calcium (mg/d)	1246 ± 413	1187 ± 459	0.305
Vitamin E (mg/d)	7.5 ± 4	7.2 ± 5	0.677
Folate (mcg/d)	384 ± 139	386 ± 110	0.310
Vitamin C (mg/d)	144 ± 70	143 ± 61	0.920
Vitamin K (mg/d)	124 ± 106	390 ± 286	< 0.0001
Cholesterol (mg/d)	290 ± 151	245 ± 117	0.005

COPD = chronic obstructive pulmonary disease

The P-values were derived using t-test

Comparing UPF consumption quintiles, participants in the highest quintile of UPF consumption, were more likely to be young, less employed, smokers, and exposed to air pollution compared to those in the lowest quintile as presented in Table 3. In terms of dietary intakes, people in the highest quintile of UPF have higher energy intake and consumed more vegetables, legumes and nuts, carbohydrates, protein, fat, cholesterol, sodium, magnesium, potassium, folate, vitamin C, vitamin K and dietary fiber, while they have lower consumption of red and processed meat compared to people with the first quintile as shown in Table 4.

**Table 3:** Study participant’s characteristics among quintiles of UPF intake score

	Q1	Q2	Q3	Q4	Q5	P-value
Age (Year)	62.27 ± 11.43	58.35 ± 12.09	54.10 ± 11.67	51.87 ± 11.21	54.88 ± 13.47	< 0.0001
Sex (Male) (%)	84.6	94.5	95.7	87.8	83.9	0.098
Married (%)	88.5	94.5	87	88.7	83.9	0.279
University Education (%)	5.8	5.5	10.1	8.2	11.3	0.726
Employment status (%)	13.5	9.1	20.3	14.3	11.3	0.003
BMI (Kg/m^2^)	25.63 ± 3.95	26.25 ± 3.47	25.81 ± 3.72	25.55 ± 4.17	26.19 ± 5.08	0.810
Physical activity (MET/Min/week)	8240.47± 9345.92	8888.82± 8421.10	10110.72± 7123.09	9794.94± 5720.93	9229.57± 7643.63	0.657
History of pulmonary disease (%)	28.1	26.7	19.4	37.8	34.3	0.511
Air pollution in habitat (%)						0.002
Urban	44.2	50.9	65.2	78.6	58.1	
Rural	26.9	23.6	24.6	12.2	16.1	
Industrial	9.6	12.7	4.3	6.1	14.5	
Hookah use (%)	1.9	1.8	2.9	6.1	6.5	0.103
Cigarette smoking (%)						< 0.0001
Current smoker	34.6	43.6	42	53.1	46.8	
Former smoker	7.7	7.3	27.5	20.4	6.5	
Never smoker	46.2	40	29	25.5	35.5	
Smoke exposure (%)	23.7	5.1	8.3	11.1	7.1	0.052
FEV1	70.74 ± 27.85	74.00 ± 29.05	78.41 ± 23.32	74.24 ± 24.27	75.20 ± 24.06	0.844
FVC	80.64 ± 18.65	82.26 ± 21.35	84.67 ± 19.78	80.89 ± 18.85	79.94 ± 17.92	0.882
FEV1/FVC	69.06 ± 16.11	70.64 ± 13.05	73.03 ± 8.06	74.26 ± 12.97	73.79 ± 11.82	0.409

COPD = chronic obstructive pulmonary disease, BMI = body mass index, FEV1 = forced expiratory volume in 1 s, FVC = forced volume capacity

P values were calculated ANOVA test for continuous variables and chi-square test for categorical variables

**Table 4:** Mean dietary intakes of study participants among quintiles of UPF intake score

	Q1	Q2	Q3	Q4	Q5	P
Energy (kcal/d)	2240.20± 623.22	2533.86± 632.20	2632.65± 612.85	2759.61± 683.36	3194.12± 727.04	< 0.0001
**Food groups (g/d)**						
Whole grains	2.90 ± 1.35	2.41 ± 1.38	3.01 ± 1.25	2.80 ± 1.44	5.63 ± 15.21	0.075
Fruit	2.31 ± 1.23	2.52 ± 1.27	2.41 ± 1.18	2.73 ± 1.26	2.87 ± 1.17	0.101
Vegetables	2.36 ± 1.33	2.66 ± 1.24	2.33 ± 1.21	2.83 ± 1.33	2.94 ± 1.27	0.031
Low-fat dairy products	2.53 ± 1.41	2.83 ± 1.11	2.50 ± 1.22	2.70 ± 1.28	2.64 ± 1.26	0.672
Legumes and nuts	2.31 ± 1.29	2.43 ± 1.12	2.27 ± 1.28	2.83 ± 1.30	2.91 ± 1.22	0.009
Red meat/processed meat	2.60 ± 1.28	2.43 ± 1.18	2.60 ± 1.16	3.04 ± 1.17	2.59 ± 1.32	0.031
Sugar-sweetened beverages	2.90 ± 1.24	2.33 ± 1.05	2.46 ± 0.95	2.84 ± 1.22	2.59 ± 1.64	0.073
**Macronutrients (g/d)**						
Carbohydrate	84.28 ± 23.85	89.75 ± 22.06	94.92 ± 19.24	98.80 ± 21.92	166.73 ± 315.68	< 0.0001
Protein	84.28 ± 23.85	89.75 ± 22.06	94.92 ± 19.24	98.80 ± 21.92	166.73 ± 315.68	0.006
Fat	57.10 ± 20.72	68.40 ± 30.03	66.78 ± 27.99	72.35 ± 25.03	93.58 ± 51.71	< 0.0001
Dietary fiber	16.67 ± 6.76	21.49 ± 9.19	19.91 ± 7.16	22.47 ± 9.71	24.60 ± 10.32	< 0.0001
**Micronutrients**						
Sodium (mg/d)	3680.49± 1777.94	3677.95± 1427.19	3772.93± 1127.96	4219.88± 1045.83	5144.35± 1787.45	< 0.0001
Magnesium (mg/d)	312.76± 145.48	368.44± 135.41	399.21± 131.24	444.73± 151.89	427.22± 165.56	< 0.0001
Potassium (mg/d)	3418.20± 1192.30	3757.63± 942.72	3915.71± 959.99	4214.55± 1006.03	5577.04± 5067.32	< 0.0001
Calcium (mg/d)	1176.97± 490.20	1131.99± 317.13	1127.38± 310.21	1152.64± 292.88	1454.48± 703.19	< 0.0001
Vitamin E (mg/d)	7.11 ± 4.06	6.87 ± 4.09	7.14 ± 7.10	7.17 ± 4.49	8.23 ± 3.68	0.599
Folate (mcg/d)	312.49± 102.74	365.84± 97.05	362.10± 118.62	381.52± 90.73	427.55± 157.00	< 0.0001
Vitamin C (mg/d)	121.93± 57.91	145.12± 55.06	136.83± 58.21	141.56± 53.65	172.18± 83.92	0.001
Vitamin K (mg/d)	176.76± 161.03	276.21± 233.62	348.77± 301.30	410.68 ± 286.65	314.31± 300.18	< 0.0001
**Others**						
Cholesterol (mg/d)	213.81± 104.68	257.85± 172.84	237.13± 93.83	274.24± 129.68	285.89± 118.88	0.016

UPF = ultra- processed food

The P-values were derived using the Analysis of Variance (ANOVA) test

Table 5 presents the multivariable-adjusted odds ratios for COPD across quintiles UPFs intake score. The odds ratios were calculated using logistic regression models and are presented for three different models. In the crude model,, consumption of UPFs was not significantly associated with the risk of COPD (OR: 0.78; 95% CI: 0.34–1.77, *P* = 0.557).After adjustment for potential cofounders in model 1 (energy intake gender and age,, and) and in model 2 (further adjusted for BMI, physical activity, hookah use and smoking status), the relationship also remained not significant (OR: 0.48; 95% CI: 0.19–1.21, *P* = 0.117 and OR: 0.36; 95% CI: 0.12–1.10, *P* = 0.074 respectively).

**Table 5:** Multivariable-adjusted odds ratios for COPD across quintiles of UPF intake score

	Q1	Q2	Q3	Q4	Q5
Crude model	1	1.09 (0.48–2.47) *P* = 0.828	0.68 (0.30–1.53) *P* = 0.351	0.51 (0.23–1.10) *P* = 0.087	0.78 (0.34–1.77) *P* = 0.557
Model 1	1	0.97 (0.42–2.27) *P* = 0.954	0.58 (0.25–1.37) *P* = 0.218	0.41 (0.18–0.97) *P* = 0.041	0.48 (0.19–1.21) *P* = 0.117
Model 2	1	0.95 (0.36–2.51) *P* = 0.911	0.44 (0.16–1.22) *P* = 0.115	0.30 (0.11–0.80) *P* = 0.017	0.36 (0.12–1.10) *P* = 0.074

The adjusted odds ratio is derived from logistic regression analysis

Model 1: adjusted for age, sex, and energy intake

Model 2: Further adjusted for BMI, physical activity, hookah use and smoking status

## Discussion

In this case-control study, we did not find any association between the consumption of UPFs and COPD. To the best of our knowledge, this is the first study to investigate this relationship.

COPD is a significant global public health concern [46]. While cessation of smoking remains the most critical public health advice for preventing COPD, research indicates that diet could also be a modifiable risk factor for impaired lung function [47]. Unfortunately, no previous studies have examined the association between processed foods and COPD. Ultra-Processed Foods (UPFs), a notable component of the Western diet [15], which is characterized by high consumption of refined grains, desserts, processed and red meats, and French fries, is generally deemed unhealthy [48]. Conversely, diets rich in fruits, vegetables, legumes, whole grains, nuts, dairy, total protein foods, seafood, and plant proteins are believed to play a protective role in the development of COPD [49], This is attributed to their high content of antioxidants (particularly vitamin C), long-chain omega-3 fats, polyunsaturated fatty acids, and dietary fiber, as well as their anti-inflammatory properties [47].

Numerous studies have explored the relationship between the Western diet, its components, and COPD. These studies consistently indicate an inverse relationship between the consumption of Western dietary components (or unhealthy diets) and COPD. For instance, a study by Varraso et al. demonstrated that over a 12-year follow-up period, the risk of newly diagnosed COPD increased with greater adherence to a Western dietary pattern It’s worth noting that this study had certain limitations, such as being conducted exclusively on American men and relying on physician diagnoses for newly diagnosed COPD without lung function test results. However, in our current study, we improved the validity and accuracy of the results by measuring lung function using a spirometry test. A study conducted in Korea by Min et al. reported increased airflow restrictions with higher consumption of soda drinks and coffee [50]. Similarly, Shi et al. found a positive association between the intake of soft drinks and COPD among adults living in South Australia [51]. However, this study’s reliance on telephone interviews for data collection and a fair response rate of 64% could potentially introduce biases. Interestingly, our study observed no significant change in the consumption of sugar-sweetened beverages within the quintiles of ultra-processed foods. This could explain the lack of significance observed in our findings compared to previous research. In other words, our study focused on the overall consumption of Ultra-Processed Foods (UPFs) rather than specific subtypes.

Numerous studies have investigated the correlation between a healthy diet and COPD, with the majority finding an inverse relationship. Steinemann et al.‘s study highlighted the protective effects of a diet rich in vegetables, fruits, nuts, and fish against age-related chronic respiratory diseases [47]. A cohort study revealed an inverse association between a prudent dietary pattern score (characterized by high intake of vegetables, fruits, whole grains, and fish) and the risk of newly diagnosed COPD A cross-sectional study also demonstrated that consumption of whole grains and fruits could positively influence FEV1 and reduce COPD prevalence [52]. However, some studies have found no connection between a healthy diet and COPD. For instance, Butler et al.‘s study found a weak association between a diet high in fruits, vegetables, and soy and the presence of cough and phlegm, but this association disappeared after adjusting for non-starch polysaccharides intake [53]. Regarding fish consumption, a major source of omega-3 polyunsaturated fatty acids in a “prudent” diet, the results from various studies have been inconsistent [54]. There was only one prospective study published on this topic, which found no association between omega-3 intake and the incidence of chronic non-specific lung disease [55].. This highlights the complexity of dietary influences on COPD and the need for further research.

In the study conducted by Fischer et al., it was found that adherence to a Mediterranean-like diet was inversely associated with the development of COPD. However, when analyzing individual components within the modified Mediterranean Diet Score (MDS), only fruit consumption was significantly linked to a reduced risk of developing COPD. This underscores the complexity of assessing dietary habits and emphasizes the importance of examining the impact of diet as a whole, rather than focusing solely on individual nutrients [56]. There seems to be no clear association between a specific food and COPD, suggesting that a more holistic approach to studying diet may provide a more comprehensive understanding of disease prevention [48]. The lack of a connection found in our study could be due to the specific foods we chose to focus on. Although our study found no association between Ultra-Processed Foods (UPFs) and COPD, studies that have observed an association suggest several mechanisms. For instance, processed meats, a component of UPFs, contain high amounts of nitrite. Nitrites produce reactive nitrogen species, which can cause nitrosative stress and potentially contribute to progressive deterioration in lung function [57]. UPFs also include refined grains, desserts, sodas, and sweets that have a high glycemic index, which can increase blood sugar levels. Hyperglycemia is associated with impaired pulmonary function [58], a primary measurement for COPD diagnosis [42]. Furthermore, both COPD and hyperglycemia are positively associated with inflammation [59, 60]. Experimental evidence suggests that foods that increase inflammation and oxidative stress can affect the pathogenesis of COPD, as COPD is associated with inflammation [61]. Sugar consumption can activate the innate immune system in the lungs and increase sensitivity to allergic airway inflammation [62]. It is also known that consumption of soft drinks can increase the risk of obesity [63], which is a risk factor for COPD [64, 65].

While several studies have suggested a potential link between UPF consumption and various health outcomes, including obesity and other diet-related noncommunicable diseases [32], the relationship with COPD is less clear. Some studies have suggested that the high levels of free sugars, saturated fats, and sodium found in UPFs could lead to systemic inflammation, a key factor in the pathogenesis of COPD [32]. However, our study did not find a significant association between UPF consumption and COPD. There could be several reasons for this. First, the effect of diet on COPD may be influenced by a range of other factors, including genetic predisposition, smoking status, and exposure to air pollution1. Second, the specific dietary patterns and food choices of our study population may differ from those in other studies, potentially influencing the observed associations. Finally, it’s also possible that the tools and methods we used to assess UPF consumption and COPD status may not have been sensitive enough to detect a potential association.

The current study is the first to examine the association of UPFs consumption with COPD risk in adults. some of the study strengths is that we adjusted the analysis to a wide range of confounding non-dietary covariates, such as age, sex, smoking, BMI, and physical activity. In addition, we matched two groups of COPD patient and healthy individuals based on age and sex. However, several limitations should be noted. Due to the cross-sectional nature of the study, we could not deduce the causal relationship. UPFs consumption was assessed using FFQ, which has potential recall bias. Also, the low number of included COPD patients, might influence on the results. Additionally, it is important to consider other factors that can highly impact COPD such as smoking or air pollution.

## Conclusion

In conclusion, our case-control study did not find any association between the consumption of ultra-processed foods (UPFs) and the risk of Chronic Obstructive Pulmonary Disease (COPD). Further studies, particularly with a prospective design, are needed to confirm these results and provide a more comprehensive understanding of the relationship between UPFs and COPD.

## Data Availability

The datasets used and/or analyzed during the current study available from the corresponding author on reasonable request.

## Abbreviations

COPD: Chronic obstructive pulmonary disease
UPFs: Ultra processed foods
FEV1: Forced expiratory volume
FVC: Forced vital capacity
FFQ: Food frequency questionnaire
BMI: Body mass index
HIV: Human immunodeficiency virus
IPAQ: International physical activity questionnaire
MET: Metabolic equivalent
ANOVA: Analysis of variance
MDS: Mediterranean diet score
